# Accumulation of catechins and expression of catechin synthetic genes in *Camellia sinensis* at different developmental stages

**DOI:** 10.1186/s40529-016-0143-9

**Published:** 2016-10-24

**Authors:** Li-Qun Zhang, Kang Wei, Hao Cheng, Li-Yuan Wang, Cheng-Cai Zhang

**Affiliations:** Key Laboratory of Tea Biology and Resources Utilization, Tea Research Institute Chinese Academy of Agricultural Sciences, National Center for Tea Improvement, Ministry of Agriculture, No. 9, Meiling South Road, Xihu District, Hangzhou, 310008 Zhejiang China

**Keywords:** *Camellia sinensis*, Catechins, Chalcone synthase, Anthocyanidin synthase, Anthocyanidin reductase, Leucoanthocyanidin reductase

## Abstract

**Background:**

Catechins are the main polyphenol compounds in tea (*Camellia sinensis*). To understand the relationship between gene expression and product accumulation, the levels of catechins and relative expressions of key genes in tea leaves of different developmental stages were analyzed.

**Results:**

The amounts of catechins differed significantly in leaves of different stages, except for gallocatechin gallate. Close correlations between the expression of synthesis genes and the accumulation of catechins were identified. Correlation analysis showed that the expressions of *chalcone synthase 1*, *chalcone synthase 3*, *anthocyanidin reductase 1*, *anthocyanidin reductase 2* and *leucoanthocyanidin reductase* genes were significantly and positively correlated with total catechin contents, suggesting their expression may largely affect total catechin accumulation. *Anthocyanidin synthase* was significantly correlated with catechin. While both *ANRs* and *LAR* were significantly and positively correlated with the contents of (−)-epigallocatechin gallate and (−)-epicatechin gallate.

**Conclusion:**

Our results suggest synergistic changes between the expression of synthetic genes and the accumulation of catechins. Based on our findings, *anthocyanidin synthase* may regulate earlier steps in the conversion of catechin, while the *anthocyanidin reductase* and *leucoanthocyanidin reductase* genes may both play important roles in the biosynthesis of galloylated catechins.

## Background

Polyphenolic compounds, which are widely distributed in plants, contribute to color (Tsuda et al. 2004) and flavor (Lea 1992) and are involved in pigment biosynthesis (Kurauchi et al. 2011), plant disease defense (Matern and Kneusel 1988) and damage caused by abiotic stress (Solovchenko and Schmitz-Eiberger 2003). Notably, polyphenols are responsible for the protective effects of plants against a variety of human diseases including cancer (Thomasset et al. 2007). Tea, which contains abundant polyphenols (Mukhtar and Ahmad 2000; Higdon and Frei 2003), is one of the most popular beverages in the world. Among the polyphenols, catechins are the major components, occupying more than 10 % of dry tea leaves (Wei et al. 2011). Catechins in tea can be separated into epicatechins (GTE) and GTE epimers. GTE include (−)-epigallocatechin gallate (EGCG), (−)-epicatechin gallate (ECG), (−)-epigallocatechin (EGC) and (−)-epicatechin (EC); while major GTE epimers include (−)-gallocatechin gallate (GCG), (−)-gallocatechin (GC) and (−)-catechin (C). The content and function vary with the individual catechin (Henning et al. 2003; Bigelow and Cardelli 2006).

**Fig. 1:**
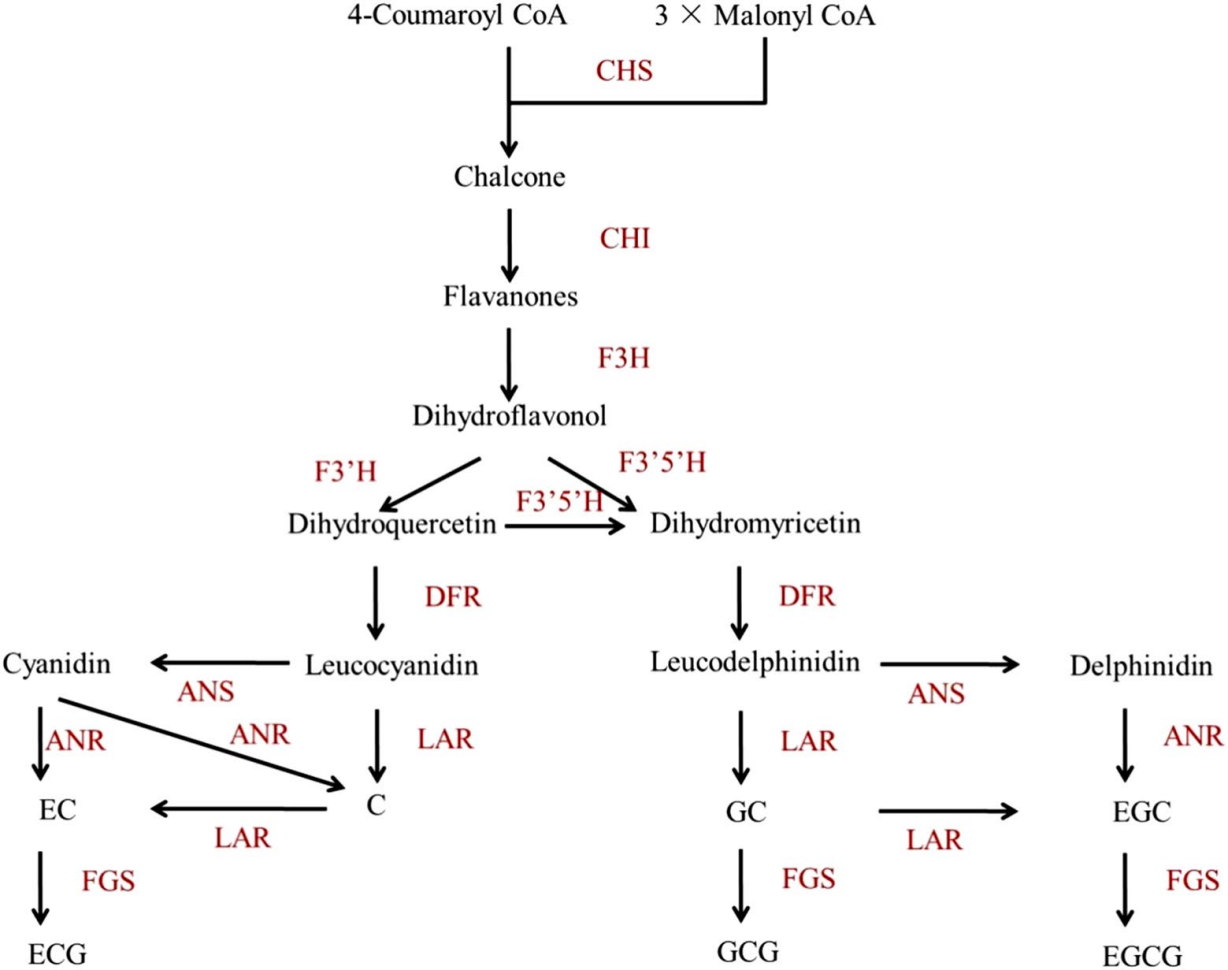
The proposed catechin biosynthetic pathway in *Camellia sinensis*. *CHS* chalcone synthase (EC 2.3.1.74); *CHI* chalcone isomerase (EC 5.5.1.6); *F3H* flavanone 3-hydroxylase (EC 1.14.11.9); *F3′5′H* flavonoid 3*′*,5*′*-hydroxylase (EC 1.14.13.88); *F3′H* flavonoid 3*′*-hydroxylase (EC 1.14.13.21); *FLS* flavonol synthase (EC 1.14.11.23); *DFR* dihydroflavanol 4-reductase (EC 1.1.1.219); *ANS* anthocyanidin synthase (EC 1.14.11.19); *ANR* anthocyanidin reductase (EC 1.3.1.77); *LAR* leucocyanidin reductase (EC 1.17.1.3); *FGS* flavan-3-ol gallate synthase (EC number not assigned)

Catechins are biosynthesized by different branches of the phenylpropanoid biosynthetic pathway (Fig. 1) (Wang et al. 2012). Chalcone synthesis is the first step in the flavonoid biosynthetic pathway, and the chalcone synthase gene (*CHS*) encodes the key enzyme for this step. A strong correlation exists between *CHS* gene expression and flavonoid content in many plants (Kamiishi et al. 2012; Morita et al. 2012; Dare et al. 2013). Sequencing of *CHS* in several plants shows that normally more than one copy is present (Jiang et al. 2006; She et al. 2013). Three highly similar copies of *CHS* in *Camellia sinensis* (*CHS1*, *CHS2* and *CHS3*) have been cloned, and their expression pattern mirrors the pattern of catechin accumulation in the leaves and stems (Takeuchi et al. 1994). Using reverse transcriptase-polymerase chain reaction (RT-PCR) and HPLC, Eungwanichayapant and Popluechai (2009) demonstrated that the accumulation of *CHS3* mRNA is higher in the shoots, where most catechins are more prominent. Expressed sequence tag analysis yielded similar results in regards to *CHS1* and *CHS2* (Park et al. 2004). Mamati et al. (2006) suggested that the expression profile of *CHS* genes in tea differs across the development stages of tea leaves. However, it is still not clear which *CHS* gene plays a more important role in affecting catechin biosynthesis. In addition to *CHS*, anthocyanidin synthase (*ANS*), anthocyanidin reductase (*ANR*) and leucoanthocyanidin reductase (*LAR*) are downstream genes in the polyphenol biosynthesis pathway (Fig. 1). *ANS* catalyzes the conversion of leucoanthocyanidins to anthocyanidins (Wilmouth et al. 2002). *ANR* is involved in the production of the flavan-3-ol monomers (such as epicatechin from anthocyanidin) (Bogs et al. 2005). While *LAR* catalyzes the conversion of leucocyanidin, leucodelphinidin or leucopelargonidin to the corresponding 2,3-trans-flavan-3-ol (Tanner et al. 2003). These genes might play important roles in determining the compositions of catechins (Saito et al. 1999; Punyasin et al. 2004; Xie et al. 2004; Wang et al. 2012) (Fig. 1). However, to what extent of these genes affect catechin compositions still remains elusive.

In this study, quantitative real time PCR (qPCR) was performed to determine the relative expression of genes involved in catechin biosynthesis, including *CHS1*, *CHS2*, *CHS3*, *ANS*, *ANR1*, *ANR2* and *LAR* in *C. sinensis* leaves at different developmental stages. Furthermore, the concentration of individual catechins was analyzed using HPLC. The aim of this study was to provide increased understanding of the regulation of catechin synthesis in the tea leaves by investigating the relationship among the accumulation of tea catechins, gene expression levels and leaf developmental stages.

## Methods

### Plant materials

Clonal tea (*C. sinensis* var. *sinensis* cultivar *Longjing 43*) was grown at the tea garden of the National Tea Research Institute of the Chinese Academy of Agricultural Sciences in Hangzhou, China. Leaf samples were collected in March 2013, when the forth leaves reached near final size. The buds, first leaves, second leaves, third leaves and completely mature leaves were collected from tea cultivar *Longjing43* for subsequently analysis. Buds, the first, second and third leaves were defined as new shoots. Part of tea samples were microwaved at high power for 1 min and then dried in an oven at 60 °C for 24 h for catechin analysis (Gulati et al. 2003). The rest collected samples were immediately frozen in liquid nitrogen and stored at −70 °C for RNA isolation.

### Catechin extraction and HPLC analysis

Catechins were extracted as described by Wang et al. (2014). Tea sample (0.2 g, dry weight) was extracted with 5 ml 70 % methanol in a water bath at 70 °C for 10 min with intermittent shaking. The supernatant was placed in a 10 ml volumetric flask, and the extraction step was repeated to reach the final volume of 10 ml. The extracts were filtered through a 0.45-μm Millipore filter before the injection was made. Each experiment was performed in triplicate.

The extracts were analyzed using an Agilent 1100 HPLC system. Separations were carried out using a Phenomenex RP-MAX 4 μm 250 mm × 4.6 mm i.d. C12 reverse phase column maintained at 40 °C, eluted at 1 ml min^−1^ with a 60 min gradient of 4–25 % gradient of acetonitrile in water containing 1 % formic acid and monitored at 280 nm (Del Rio et al. 2004).

The standard chemicals, including catechin (C), epicatchin (EC), gallocatechin (GC), epigallocatechin (EGC), Epicatechin gallate (ECG), gallocatechin gallate (GCG), epigallocatechin gallate (EGCG) were purchased from Sigma Chemical Company (St. Louis, MO, USA).

### Quantitative real time PCR (qPCR)

Total RNA from the samples was extracted using the SV Total RNA Isolation System (Promega Corporation, USA) as described in the technical manual. The integrity and quality were checked by determining absorbance using the NanoDrop spectrophotometer ND-1000 (NanoDrop Technologies Inc., USA). First strand cDNA was synthesized from 320 ng of total RNA and oligo (dT) primer using the PrimeScript II 1st Strand cDNA Synthesis Kit (TAKARA, Japan) as described in the product manual. The cDNA was used immediately or stored at −20 °C until used for the PCR test.

Primers for PCR (Table 1) were designed using Primer 3.0 according to the sequences of *CHS1* (Accession No. D26593), *CHS2* (Accession no. D26594), *CHS3* (Accession no. D26595), *ANS* (Accession no. GAAC01051116), *ANR1* (Accession no. GU944768), *ANR2* (Accession no. JN024667) and *LAR* (Accession no. EF205148). The PCR analysis was carried out using the ABI PRISM7500 Fast Real-Time PCR System as described in the SYBR*Premix* Ex Taq™ (perfect real time) (TAKARA, Japan) kit manual. *18S rRNA* from *C. sinensis* was taken as an internal control gene. Three replications were taken for each experiment.

**Table 1:** Primers of catechin biosynthetic genes for real-time PCR analysis in *Camellia sinensis*

Gene	Accession no.	Primer sequence 5′−3′
*CHS1*	D26593	Forward: CAGAGCACGTACCCGGATTATTAC
		Reverse: GGTACTTCAACCACAACCATGTCC
*CHS2*	D26594	Forward: CATTACTAACAGCGAGCATAAAACG
		Reverse: TAGTTTTGGGACTTCAACAACCAC
*CHS3*	D26595	Forward: CCTCTTCCTAGCTAGCACATACCA
		Reverse: TGCTCGCTATTAGTAATCCGAAAGT
*ANS*	GAAC01051116	Forward: TGGGAAGACTATTTCTTCCACCTT
		Reverse: TTCTAGTCGGCCTTCTTCTAGTCC
*ANR1*	GU944768	Forward: CCTTCTAGCACTAAAGGGTTCAGG
		Reverse: TGACTACTCCTTGAATTGCTGGTT
*ANR2*	JN024667	Forward: TCACAGGGAATGAATTCCTCATAG
		Reverse: GGAACATTGTACTGGGGGTATCTT
*LAR*	EF205148	Forward: TTTTGTTGCAGGCTCTGATATAGG
		Reverse: ATTATATTCACTGCTGCTGCTGCT
*18S rRNA*	AB120309.1	Forward: TCTCAACCATAAACGATGCCGACCAG
		Reverse: TTTCAGCCTTGCGACCATACTCCC

### Statistical analysis

Results were analyzed by one-way analysis of variance (ANOVA) and statistical differences were examined by the Fisher’s least significant difference (LSD) test. Differences at p < 0.05 were considered to be significant. Correlations analysis was determined using SAS 9.0 software, and charts were plotted using Sigma Plot 12 software.

## Results

### Catechin analysis by HPLC

**Fig. 2:**
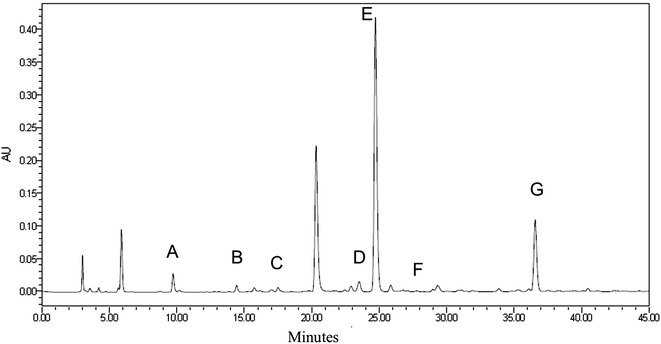
Chromatogram of the catechin components in tea. Results from a representative HPLC experiment are shown. The individual peaks represent *A* (gallocatechin; GC), *B* (epicatechin gallate; EGC), *C* (catechin; C), *D* (epicatechin; EC), *E* (epigallocatedchin gallate; EGCG), *F* (gallocatechin gallate; GCG), and *G* (epicatechin gallate; ECG)

**Fig. 3:**
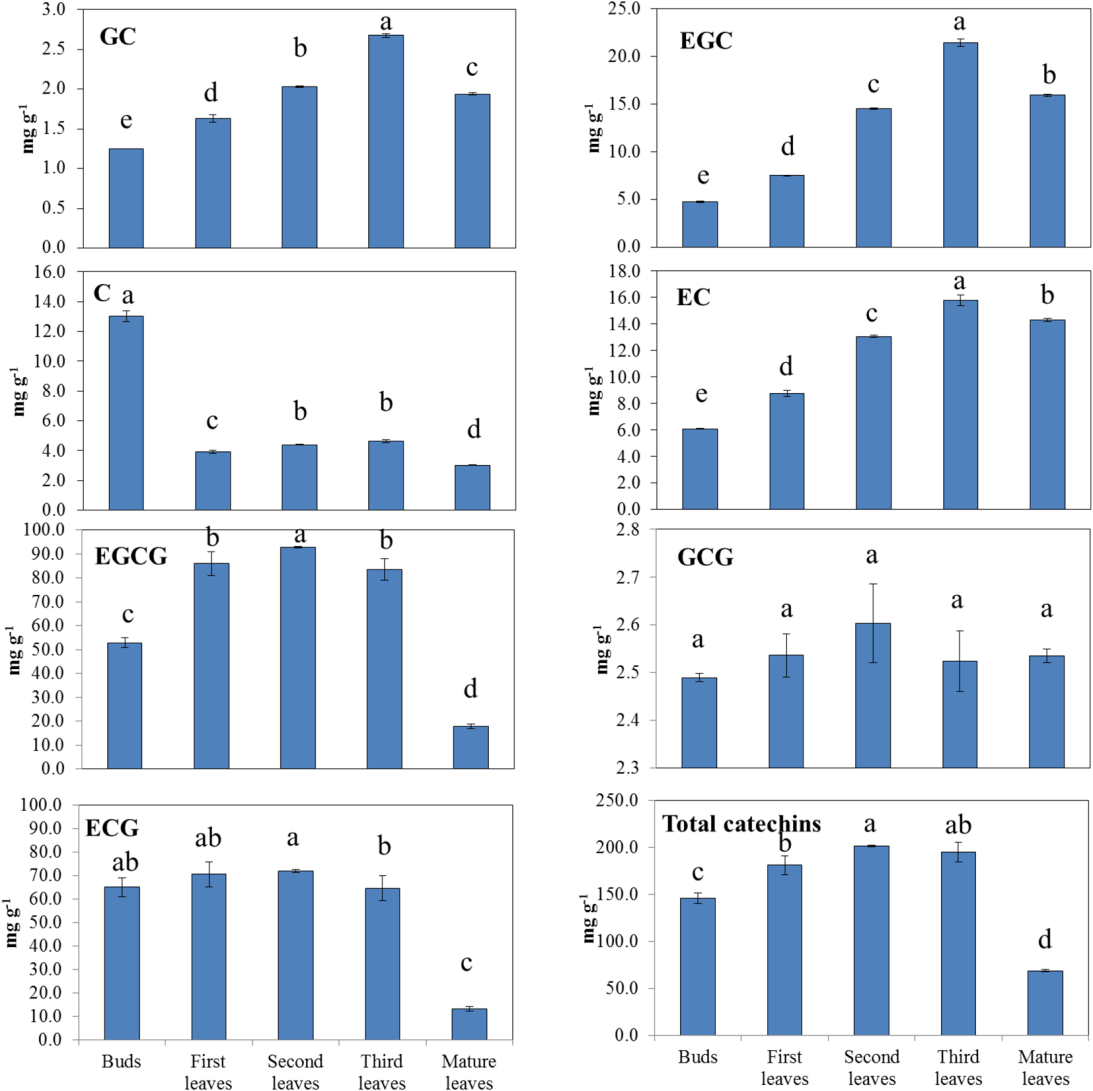
The changes of catechin fractions and total catechins in tea leaves of different stages. *Lower case letters* on the *upper right corner*: Statistically significant difference as determined by Fisher’s least significant difference test. *Same letters* in each *column* mean there is no significant difference in the same catechin in different developmental stage leaves, otherwise, significantly different (p = 0.05)

Good separation was achieved with peaks identified as the 7 individual tea catechins (Fig. 2). The order of peaks was GC, EGC, C, EC, EGCG, GCG and ECG. The catechin contents in different leaf stages are shown in Fig. 3. It could be found that EGCG and ECG had the highest concentrations, totally occupied about 80 % of total catechin contents. While the contents of GC and GCG were relatively low, ranging from 1.25 to 2.68 and 2.49 to 2.60 mg/g respectively. The amounts of all catechins differed significantly at different developmental stages, with the exception of GCG, which remained relatively constant at all stages (p = 0.180). The second leaves contained the highest total catechin content, and the individual catechins, with the exception of GCG, were most concentrated in the new shoots. The mature leaves had the lowest C, ECG, EGCG and total catechins. Among the new shoot samples, a general increase of the non-galloylated catechins was observed with increasing age of the leaves, with the third leaves having the highest concentration. The pattern of galloylated catechins in the new shoots was similar, except that the highest concentration appeared in the second leaves. According to the biosynthetic pathways of flavan-3-ols in *C. sinensis* leaves (Ashihara et al. 2010), galloylated catechins are synthesized through non-galloylated catechins with the help of flavan-3-ol gallate synthase (FGS). Hence, we deduce that the distinction found in this experiment may be caused by a variation of FGS activities. It was also worth noting that the highest C content was observed in the buds, with a sharp decrease at the rest stages, which was consistent with our previous findings (Wei et al. 2011).

### Expressions of catechin synthetic genes

**Fig. 4:**
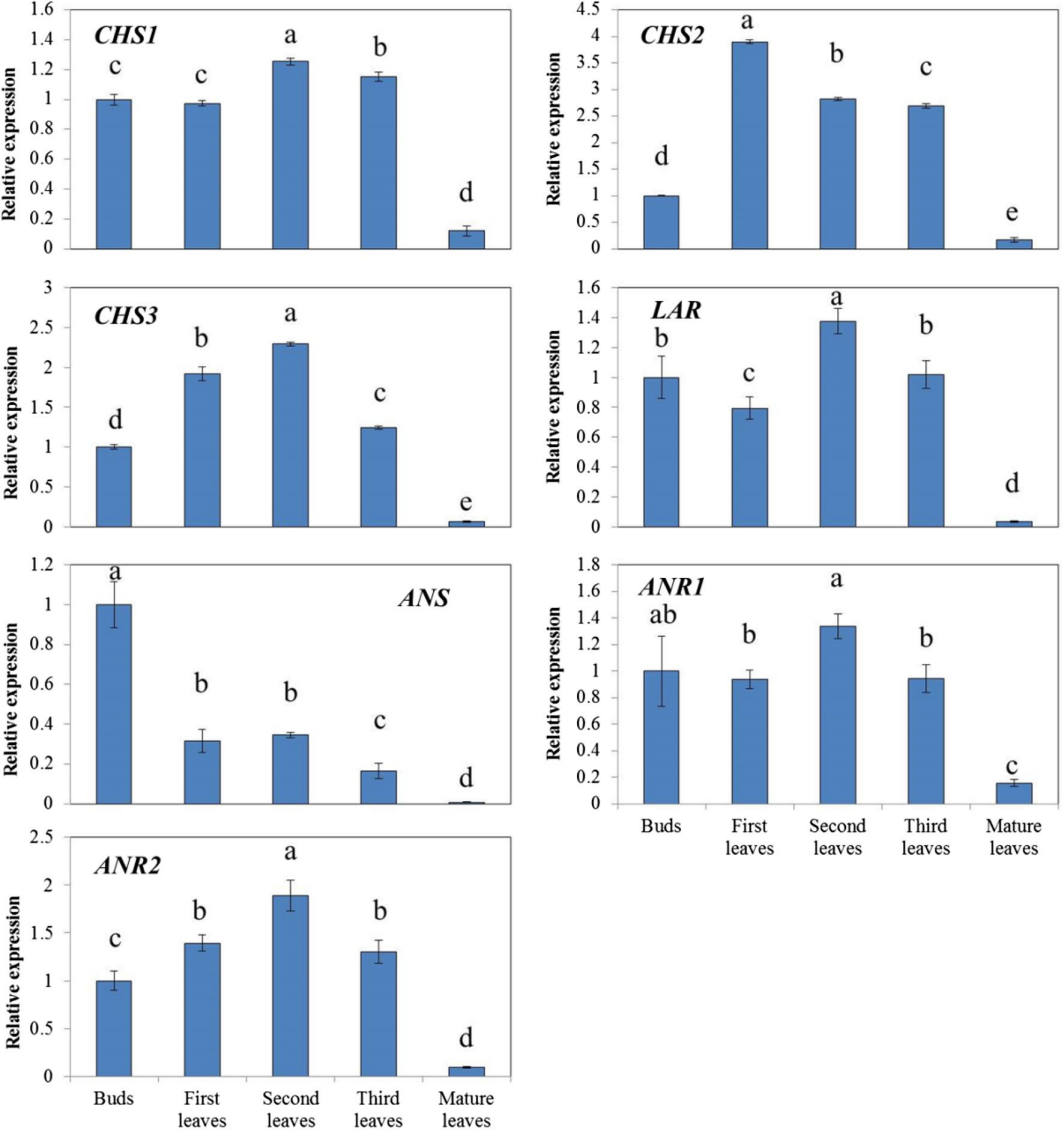
Relative expressions of catechin synthetic pathway genes in tea leaves of different stages. The relative expressions of the CHS, ANS, ANR and LAR genes at the bud stage, the new shoot stage (1st, 2nd and 3rd leaves), and the mature leaves stage were shown. Expression of each gene was standardized to the expression of 18s rRNA and normalized to 1 at the bud stage. Results represent averages ± SD of three plants tested in triplicate

To investigate the developmental expression patterns of catechin synthetic genes, *CHS1*, *CHS2*, *CHS3*, *LAR*, *ANS*, *ANR1* and *ANR2* in *C. sinensis*, qPCR was performed using gene-specific primers to measure expression levels (Fig. 4). cDNA samples prepared from leaves at different developmental stages were used as template. Transcript levels of all of these genes were at least 5-fold higher in young leaves than in mature leaves. Furthermore, the relative expression levels differed at different developmental stages. The expression of *CHS2* showed the largest variation in the 3 *CHS* genes, ranging from 0.172 in the mature leaves to 3.901 in the first leaves. Whereas *CHS1* and *CHS3* exhibited relatively less variations, ranging from 0.122 to 1.254 and 0.066 to 2.293 respectively. Moreover, the expressions of *CHS1* and *CHS3* peaked at the second leaf stage, which was different from that of *CHS2*.

The expression of *LAR* fluctuated dramatically during the developmental stage. Down by 20 % at the first leaf stage, *LAR* showed a sharp rise afterward, reaching its peak at the second leaf stage. Then it was followed by a significant fall in the third leaves. The expression of *ANR1* and *ANR2* showed a similar pattern to *LAR* during the developmental stages. Both of them reached their peaks at the second leaf stage while *ANR2* showed larger variation. It is also worth noting that the expression pattern of *ANS* was largely different from the other genes. *ANS* showed a general downward trend during the developmental stage, with its peak at the bud stage and the bottom at the mature leaf stage.

### Correlation analysis of catechin contents and relative gene expressions

The correlation between catechin contents and relative gene expressions was analyzed in Table 2. Correlation analysis showed that *CHS1* was highly significantly and positively correlated with ECG and total catechin contents, and significantly correlated with EGCG, indicating it might play a more important role in influencing catechin accumulation than *CHS2* and *CHS3*. Furthermore, both *ANR1* and *ANR2* were significantly correlated with total catechins, EGCG and ECG, suggesting *ANRs* are essential for EGCG and ECG biosynthesis. On the other hand, *ANS* showed no significant correlation with EGCG and ECG, but was significantly and positively correlated with C, indicating its potential role in C biosynthesis. It is also worth noting that *LAR* was not significantly correlated with C and GC, but showed positive relationship with ECG and total catechins, suggesting the possible conversion of C to EC in tea plants.

**Table 2:** The correlation between catechin contents and relative gene expressions

	GC	EGC	C	EC	EGCG	GCG	ECG	Total catechins
*CHS1*	0.134	−0.069	0.265	−0.174	0.916*	0.224	0.968**	0.964**
*CHS2*	0.222	−0.009	−0.312	−0.029	0.919*	0.378	0.775	0.854
*CHS3*	0.036	−0.150	−0.087	−0.158	0.937*	0.579	0.871	0.896*
*LAR*	0.081	−0.082	0.309	−0.171	0.853	0.329	0.917*	0.906*
*ANS*	−0.714	−0.761	0.959*	−0.847	0.082	−0.447	0.487	0.176
*ANR1*	−0.016	−0.190	0.289	−0.262	0.879*	0.351	0.948*	0.913*
*ANR2*	0.146	−0.044	0.017	−0.091	0.965**	0.509	0.921*	0.961**

* Represents significant difference at 95 % probability level (p < 0.05)

** Represents significant difference at 99 % probability level (p < 0.01)

## Discussion

The concentration of 7 individual catechins in tea leaves, including C, GC, EC, EGC, ECG, EGCG and GCG were determined in the current study, among which EGCG and ECG were found to be the highest fractions and GC and GCG were the lowest. With the exception of GCG, which did not differ among developmental stages, the highest concentrations of individual catechins were in new shoots. Furthermore, for new shoots, a general increase in the levels of individual catechins and total catechins was observed with increasing age of the leaves with an exception of C, whose concentration is highest in the buds. These results are similar with the results of two other studies (Eungwanichayapant and Popluechai 2009; Wei et al. 2011). In contrast, the total catechins in *C. sinensis* var. *sinensis* cultivar *Zhenong 139* were found to slightly decline with increasing age of the leaves (Mamati et al. 2006). These differences might be explained by genetic variations or differences in the methods of cultivation, post-harvest treatment, environment or agricultural practices (Eungwanichayapant and Popluechai 2009; Wei et al. 2011).

Many studies have shown that gene expression and product concentration are associated with phenotypic changes. Tea catechins are considered to be synthesized through flavonoid pathway and stored in the vacuole (Suzuki et al. 2003; Rani et al. 2009). As major enzymes involved in catechin oxidation such as polyphenol oxidase (PPO) and peroxidase (POD) are present in latent forms that require activation or in different subcellular compartments, catechin degradations only occur after senescence or stresses that disorganized the cell and initiated decompartmentalization (Pourcel et al. 2007). Therefore, the expressions of catechin synthetic genes are considered to be the major factors affecting catechin contents in tea (Saito et al. 1999; Punyasin et al. 2004; Xie et al. 2004; Wang et al. 2012). In this study, the expression levels of catechin synthetic pathway genes in tea leaves at different developmental stages were analyzed. General increases of both the catechin concentrations and the expression of synthetic genes were found in new shoots, indicating their potential correlations. High correlation coefficients were identified between total catechins and *CHS1* (0.964, p < 0.01), *CHS2* (0.854) and *CHS3* (0.896, p < 0.05) (Table 2), indicating the importance of *CHS* in catechin biosynthesis. Furthermore, according to the changing pattern and correlation coefficients, *CHS1* seems to play a more important role in affecting catechin accumulation. In fact, the expression of *CHS1* was much higher than *CHS2* and *CHS3* in tea leaves based on RNA-seq analysis (data not shown). Therefore, *CHS1* should be paid more attention to in future studies.

In terms of catechin individuals, C had a different expression profile than the other catechins, with peak concentration in the buds, which was consistent with our previous study (Wei et al. 2011). Moreover, *ANS* was also the highest in the bud stage, suggesting the possibility that C conversion is predominantly regulated by *ANS*. Previously, it was considered that C was converted from leucocyanidin by *LAR* (Ashihara et al. 2010). However, from the present results, no significant correlation between the expression of *LAR* and C content was found (Table 2). Besides, high expression of *LAR* seems to lead to a high accumulation of ECG and total catechins. This raised the question about the role of *LAR* in tea catechin biosynthesis. Stafford (1990) found *LAR* is able to convert leucocyanidin to C with an epimerase, and then convert C to EC. While, transformation of *CsLAR* into purple tobacco plants leads to a significant decrease in anthocyanin, with accumulation of 13.7- and 7.2- fold more EC and C, respectively (Pang et al. 2013). Our results are consistent with these studies and indicate that C might be involved in the conversion of leucocyanidin to EC as an intermediate. Moreover, according to Pang’s study, *CsANR1* and *CsANR2* were able to convert cyanidin to a mixture of EC and C. Therefore, it is hypothesized that compared to the conversion of leucocyanidin to C by *LAR*, the conversion of leucocyanidin to cyanidin catalyzed by *ANS*, and then to C by *ANR*s is more important for C accumulation (Fig. 1).

On the other hand, EC, EGC, EGC and EGCG are also known to be synthesized through enzymatic reactions catalyzed by *ANS* and *ANR* (Wang et al. 2012). While the accumulation patterns of these products had similar trends with *ANRs*. Furthermore, correlation analysis showed both *ANR1* and *ANR2* were significantly and positively correlated with ECG and EGCG (Table 2). Therefore, the expressions of *ANRs* may comprise a rate-limiting step in ECG and EGCG productions.

## Conclusions

In conclusion, this study demonstrated that close correlations between the expressions of synthesis genes and the accumulation of catechins were present in tea plants. Based on our findings, *CHS1* is more important in catechin biosynthesis than *CHS2* and *CHS3*. *ANS* may regulate the conversion of C, while both *ANRs* and *LAR* may play important roles in the biosynthesis of EGCG and ECG.

## Abbreviations

ANR: anthocyanidin reductase
ANS: anthocyanidin synthase
C: (+)-catechin
CHI: chalcone isomerase
CHS: chalcone synthase
DFR: dihydroflavanol 4-reductase
EC: (−)-epicatechin
ECG: (−)-epicatechingallate
EGC: (−)-epigallocatechin
EGCG: (−)-epigallocatechingallate
F3H: flavanone 3-hydroxylase
F3′H: flavonoid 3′-hydroxylase
F3′5′H: flavonoid 3′,5′-hydroxylase
FGS: flavan-3-ol gallate synthase
FLS: flavonol synthase
GC: (−)-gallocatechin
GCG: (−)-gallocatechingallate
LAR: leucocyanidin reductase
RT-PCR: reverse transcriptase-polymerase chain reaction
